# Experimental Validation of Microwave Tomography with the DBIM-TwIST Algorithm for Brain Stroke Detection and Classification

**DOI:** 10.3390/s20030840

**Published:** 2020-02-04

**Authors:** Olympia Karadima, Mohammed Rahman, Ioannis Sotiriou, Navid Ghavami, Pan Lu, Syed Ahsan, Panagiotis Kosmas

**Affiliations:** Faculty of Natural and Mathematical Sciences, King’s College London, Strand, London WC2R 2LS, UK; mohammed.3.rahman@kcl.ac.uk (M.R.); ioannis.sotiriou@kcl.ac.uk (I.S.); navid.ghavami@kcl.ac.uk (N.G.); pan.lu@kcl.ac.uk (P.L.); syed.s.ahsan@kcl.ac.uk (S.A.)

**Keywords:** microwave tomography, stroke detection, DBIM

## Abstract

We present an initial experimental validation of a microwave tomography (MWT) prototype for brain stroke detection and classification using the distorted Born iterative method, two-step iterative shrinkage thresholding (DBIM-TwIST) algorithm. The validation study consists of first preparing and characterizing gel phantoms which mimic the structure and the dielectric properties of a simplified brain model with a haemorrhagic or ischemic stroke target. Then, we measure the S-parameters of the phantoms in our experimental prototype and process the scattered signals from 0.5 to 2.5 GHz using the DBIM-TwIST algorithm to estimate the dielectric properties of the reconstruction domain. Our results demonstrate that we are able to detect the stroke target in scenarios where the initial guess of the inverse problem is only an approximation of the true experimental phantom. Moreover, the prototype can differentiate between haemorrhagic and ischemic strokes based on the estimation of their dielectric properties.

## 1. Introduction

Brain stroke is a medical condition that is caused by a blocked (ischaemic) or burst (haemorrhagic) brain vessel, resulting in damage and necrosis of the affected brain tissue. The vast majority of the reported cases, almost 87% of them, are ischaemic. According to the American Heart Association, brain stroke is one of the leading causes of death in the USA [1]. There are different windows of treatment, depending on the type of the stroke and the area of brain that is injured. Determining the stroke type as early as possible is critical, as the wrong or delayed treatment could be lethal [2]. Detection and localization relies on magnetic resonance imaging (MRI) and, more commonly, on computed tomography (CT) scans. While both MRI and CT are accurate and reliable methods, neither of them are truly portable and thus ready to be used widely inside an emergency vehicle for detecting strokes as early as possible. Moreover, MRI is expensive, and CT is associated with health risks due to ionized radiation [3]. These challenges motivate the development of alternative approaches, which aim to be fast, safe, portable and cost-effective.

Microwave imaging (MWI) can potentially offer these advantages, and has thus emerged as a promising alternative for diagnosing cardiovascular diseases which could be used prior to MRI or CT scans [4]. Efforts to develop MWI systems for medical diagnostics go back almost forty years, with several review papers, book chapters, and special issues reporting recent developments [5,6,7]. Amongst a large body of research in medical microwave imaging, which cannot possibly be fully reviewed here, we note that various experimental systems have been developed for breast cancer detection [8,9,10,11,12,13,14], and more recently for stroke monitoring and detection [15,16,17]. Prototypes based on machine learning have also been developed and clinically tested [18]. With regards to microwave tomography specifically, for brain imaging, a study by Hopfer et al. using a microwave scanner of 177 antennas presented successful experimental reconstruction results for stroke detection and brain monitoring [19]. In addition, the design of a microwave tomography (MWT) scanner for brain imaging has been analysed in References [20] and [21] to determine suitable frequencies and properties of the coupling medium, as well as to optimize the antenna array design.

In a MWT system, an array of antennas transmits electromagnetic (EM) waves which penetrate into the tissue, and receives the scattered field [22]. The MWT algorithm reconstructs a map of the dielectric properties of the imaged region to locate a target with unknown dielectric properties, by solving an inverse and ill-posed EM scattering problem [23,24]. MWI techniques for medical applications rely on the dielectric contrast between healthy and diseased tissues, which depends on their water content [25]. The dielectric properties of human tissues have been extensively studied and reported in References [25,26]. The head’s dielectric properties have been measured in References [27] and [28]. For the case of ischaemia, the measurements in [29] reported that the properties of an ischaemic vessel in the brain can vary from −10% to −25% relative to the dielectric properties of a healthy brain.

MWT methods are challenged by the high heterogeneity of the human body [30] as well as the increased computational cost that arises from the non-linearity of the problem and the non-uniqueness of the solution [31]. Based on these considerations, we have developed previously a robust algorithm based on the distorted Born iterative method (DBIM) and the two-step iterative shrinkage/thresholding (TwIST) solver for microwave breast imaging [32,33], which was then incorporated successfully in a prototype tested in experiments with simple cylindrical targets [34,35,36] and showed advantages in comparison with other DBIM linear solvers like conjugate gradient method for least squares (CGLS) or iterative shrinkage/thresholding (IST) [32,33,36].

Taking into account the challenges of MWT systems and the medical requirements for brain stroke detection and differentiation, the aim of this paper is to validate experimentally DBIM-TwIST and our prototype for stroke imaging using brain tissue and stroke mimicking phantoms. To this end, we present for the first time reconstruction results across a wide frequency range, which demonstrate the potential of differentiating between stroke types based on a quantitative estimation of their dielectric properties. Moreover, we show our method’s robustness to differences in the dielectric properties of the background medium assumed in our inverse phantoms relative to the phantom used in the experiment. We therefore believe that these experimental results provide further evidence of MWT’s potential to detect strokes based on microwave images produced by a carefully designed system and algorithm.

The remainder of the paper is organized as follows: Section 2 presents the methodology for phantoms preparation and characterization, the experimental data acquisition process, and a summary of our DBIM-TwIST algorithm. Section 3 presents reconstruction results for haemorrhagic and ischaemic mimicking targets, and for phantoms with different dielectric properties than the initial guess used in the inversion. Finally, Section 4 summarises our findings and discusses our future work towards developing a complete MWT system for brain stroke imaging.

## 2. Materials and Methods

### 2.1. Phantoms Preparation and Characterization

Experimental testing of MWT systems is critical in order to assess their potential for clinical applications. To this end, phantoms that mimic the dielectric and structure properties of the human head and brain provide efficient, easy to fabricate, and low-cost testbeds for experimental validation prior to clinical trials. Gelatine emulsions made of oil, water and gelatine are popular recipes for preparing such phantoms, as they are cheap and easy to alter, but can suffer from dehydration. McDermott et al. have proposed an alternative method of constructing solid phantoms made of ceramic and carbon powder, in which solid phantoms were fabricated with polyurethane, graphite, carbon black and isopropanol, to mimic the dielectric properties of the brain and the average properties of the layers surrounding the brain [37]. Solid phantoms are electrically and mechanically stable as well as reusable, but are more difficult to fabricate, time consuming, more expensive than the liquid phantoms, and not easy to modify for additional adjustments in properties or geometry. In Reference [38], an alternative method of 3D printed moulds filled with fluid TX100 salted water mixtures that can mimic the dielectric properties of head tissues was proposed. Those phantoms are cheap, easy to alter, reusable and stable. They require, however, plastic mould materials which can cause disturbances in the scattered signals [39].

**Figure 1:**
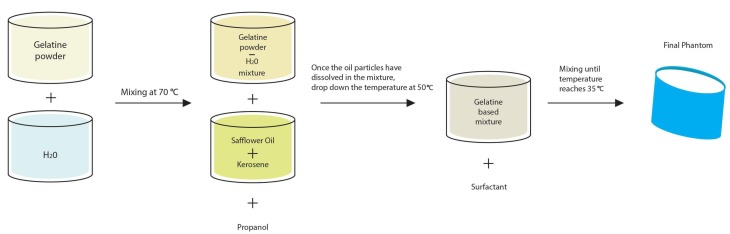
Schematic representation of the phantoms’ preparation process.

As the aim of this work is to validate DBIM-TwIST in more realistic, yet simplified cases for brain stroke detection and classification, we have constructed simple phantoms based on the materials and processes proposed in Reference [40] for breast phantoms. Using different gelatine-oil concentrations compared to those for breast tissues, we fabricated tissue-mimicking materials with specific dielectric properties that mimic average brain tissue, cerebrospinal fluid (CSF), blood and ischaemia. The process is illustrated in Figure 1. First, a water-gelatine mixture is prepared and mixed at 70 ${}^{{}^{\circ}}$C, until the gelatine particles are fully dissolved into water and the mixture is transparent. Propanol is added to deal with the creation of air bubbles on the surface. Once heated to 70 ${}^{{}^{\circ}}$C, we add a 50% kerosene-safflower oil solution into the water-gelatine mixture and stir at the same temperature, until the emulsion has an opaque white colour and the oil particles are fully dissolved. We keep stirring the mixture until the temperature drops to 50 ${}^{{}^{\circ}}$C, when we add the surfactant. Finally, we pour the prepared mixture into the mould when it reaches 35 ${}^{{}^{\circ}}$C, and let it set overnight before we conduct any measurements.

**Figure 2:**
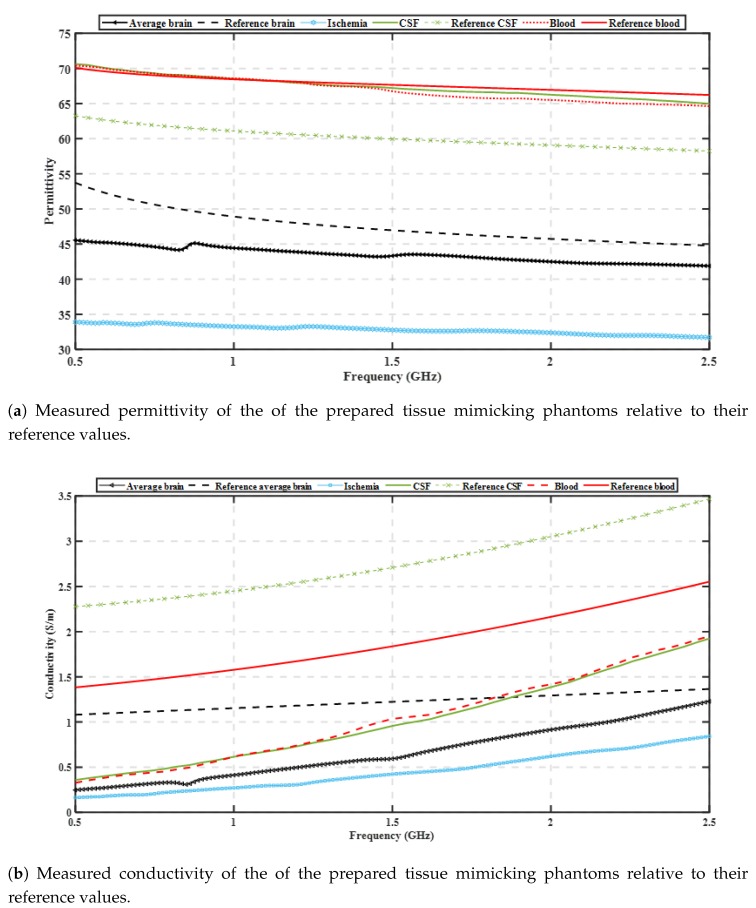
Dielectric properties of the fabricated tissue mimicking phantoms relative to reference values taken from [25] (to the authors’ knowledge, there is no literature regarding the dielectric properties of ischaemia in a wide frequency range). Same type of tissues are presented with the same colour.

Table 1 presents the concentrations of the materials used for the phantoms mimicking different brain tissues used in our experiments. We fabricated the four tissue-mimicking phantoms, using as reference the properties reported in Reference [25]. The in-vivo measurements in [29] reported that properties of an ischaemic vessel in the brain can vary from −10% up to −25% relative to the dielectric properties of healthy brain tissue at 1.0 GHz. Therefore we chose to prepare ischaemic phantoms with the maximum reported difference of −25% relative to the properties of the brain phantom. For the experimental testbeds it is assumed that, due to similar dielectric properties of CSF and blood, the same gelatine mixture can be used to mimic the two different tissues. Figure 2 presents the measured dielectric properties of the prepared phantoms for brain, CSF, blood and ischaemia, respectively. The measurements are conducted using Keysight’s dielectric spectroscopy kit, over a 0.5–2.5 GHz frequency range at different points of the phantoms. The plots in Figure 2 show very good agreement with reference data for the dielectric constant, but quite lower conductivity values for our phantoms. As MWT relies mostly on contrast in dielectric constant, these discrepancies are not critical for this initial investigation.

**Figure 3:**
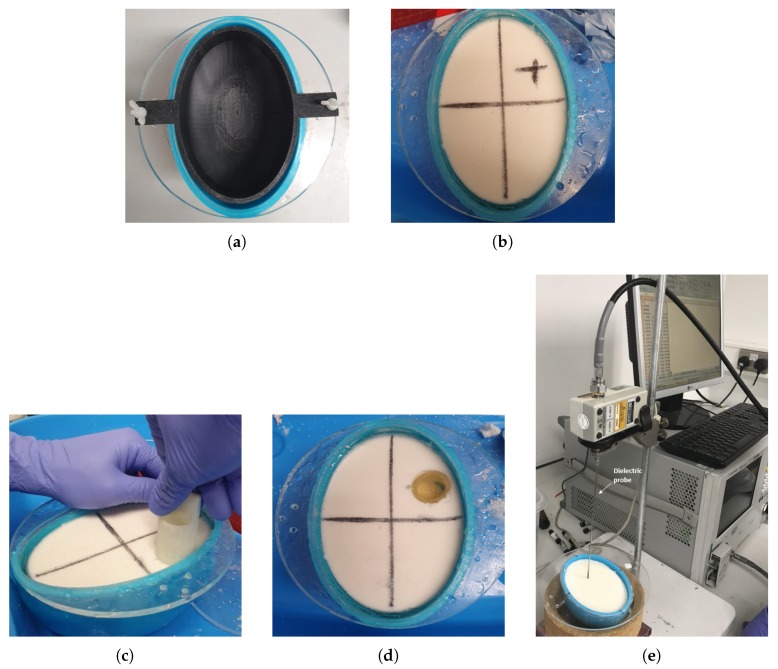
Summary of the head model construction: (**a**) Cylindrical 3-D printed mould to form the cerebrospinal fluid (CSF) and average brain layers; (**b**) The two-layer model after CSF and average brain phantoms are poured into the moulds (the CSF is a thin, transparent layer just inside the blue mould); (**c**) Creating a hole in the phantom to insert the stroke-like target; (**d**) Final two-layer phantom with a target of blood-mimicking phantom; (**e**) Setup with Keysight’s slim form probe to measure the dielectric properties of the phantoms.

After preparation, phantoms are placed in elliptical plastic acrylonitrile butadiene styrene (ABS) moulds which aim to mimic the brain’s shape and multi-layer structure, as shown in Figure 3a,b. We first pour the CSF phantom in the outer layer of 5 mm thickness. After this is solidified, we extract the inner mould and fill the remaining cavity with the brain phantom. For a one-layer model without the CSF, we simply extract the inner mould and pour the phantom in the outer mould directly. We use an additional cylindrical mould to create a hole (diameter = 30 mm) in the phantom (Figure 3c), which can be filled with the phantom mimicking either blood or ischaemia (Figure 3d), emulating the two cases of brain stroke termed as h-stroke for haemorrhagic and i-stroke for ischaemic, respectively.

To further investigate the change in the dielectric properties over time, we prepared two additional brain phantoms and measured their real and imaginary part of permittivity on day 1 and day 6 after their preparation. Table 2 shows that, over a period of 6 days, the dielectric properties increased 10–15% compared to their initial values. This is expected as the water particles have dispersed over time resulting in higher dielectric properties.

### 2.2. Setup and Data Acquisition Process

**Figure 4:**
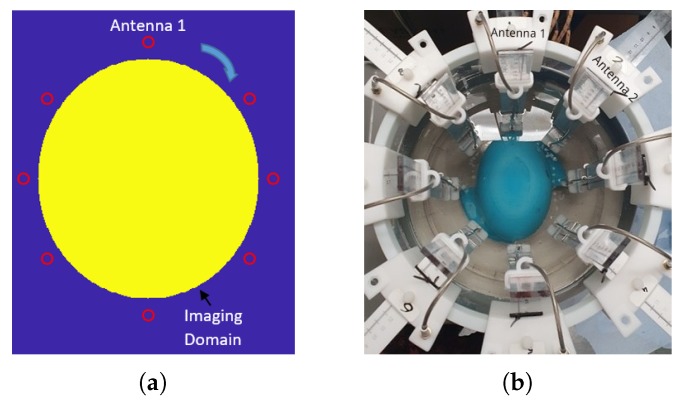
(**a**) Schematic of the simplified “initial guess” model for the inversion (the ellipsoid’s axes are 153 mm and 112 mm long); (**b**) The hardware system prototype.

Figure 4b presents the experimental setup, which is used for conducting the measurements. It consists of a 300 mm diameter cylindrical tank surrounded by an absorber (ECCOSORB MCS) and enclosed with a metallic shield to decrease surface waves propagating into the perimeter of the tank. The tank is filled with a 90% glycerol-water mixture to improve impedance matching with the phantom and widen the antenna operating frequency range [34]. An eight-antenna elliptical array is immersed inside the tank, and the length of the ellipsoid’s axes are 153 mm and 112 mm. From a theoretical point of view, the choice of the number of antennas is defined as:

(1) M=2βα,

where α is the radius of the reconstruction domain and β is the wave-number [41]. Taking into account our first working frequency of 0.5 GHz, *M* is approximately equal to 15. However, due to our hardware limitation of the 8-port VNA and our previous experimental work which has showed good results using eight antennas in our MWT prototype, we chose eight antennas to simplify the experiment and the data acquisition process [34]. The experiments presented in this study use spear-shaped patch monopole antennas which operate efficiently in the range of 0.5–2.0 GHz and their design was presented in Reference [42]. This range satisfies the recommendation by the theoretical approach in Reference [20], which suggests that a working frequency up to 1.5 GHz can achieve the optimal trade-off between the required resolution and incident power needed for head imaging applications. Antennas are installed into vertical and horizontal rulers that enable us to adjust their height and the array dimensions.

Phantoms are placed in the bottom of the tank as shown in Figure 4b, and measurements of the S-parameters of the scattered signals are conducted in a frequency range of 0.5–2.5 GHz using an eight-port vector network analyzer (VNA) from Keysight. The eight antennas act both as transmitters and receivers, creating an 8×8 scattering matrix which is fed into DBIM-TwIST. The linear integral equation is discretized iteratively for the entire 8×8 transmit-receive pairs. Overall, two sets of measurements are performed for every imaging scenario. The first set of measurements is performed for the phantom without a target (the “no target” scenario), and then the process is repeated for the phantom which includes the 30 mm diameter h-stroke or i-stroke target (“target” scenario). The target is placed in the upper-right section of the phantom between antennas 1 and 8. Following this process, the S-parameters for “no target” and “target” scenarios are processed by the DBIM-TwIST algorithm [32], which is reviewed in the next subsection.

### 2.3. Implementation of the DBIM-TwIST Algorithm

To reconstruct the dielectric properties of the phantoms, we apply our formerly developed DBIM-TwIST algorithm, which has been widely validated by our previous studies in different numerical [32,33] and experimental scenarios involving extended cylindrical targets [34,35,36]. In DBIM, the nonlinear scattering problem which arises from the integral equation of the scattered electric field is discretized under the Born approximation as follows:

(2) $$\begin{array}{cc} E_{s}\left(r_{n},r_{m}\right) & \approx \iota \omega \int_{V}E_{b}\left(r,r_{m}\right)E_{b}\left(r,r_{n}\right)O\left(r\right)dr \end{array},$$

where $E_{s}$ and $E_{b}$ are the scattered and background fields respectively, and $r_{n}$, $r_{m}$ denote transmitter and receiver positions, respectively. The contrast function O(r) is the difference between the complex permittivity of the known background and the unknown region. The equation is solved iteratively and requires the solution of a linear but ill-posed problem at each DBIM iteration [23]. The background properties are updated at each DBIM iteration with the current solution until the residual error is minimised, and this yields the reconstruction of the dielectric properties of the imaging domain. This is done by discretizing the above integral equation as,

(3) Ax=y,

where *A* is an M-by-K propagation matrix, with M the number of transmit-receive pairs in the antenna array and K the number of elements in the discretization inside the reconstruction domain, while *y* is the M-by-1 vector of the scattered fields recorded at the receivers. The K-by-1 vector *x* is the unknown dielectric properties contrast function, which is then added to the previous background profile to generate the new background which is used for the next iteration [23]. Our algorithm solves this linear problem with the TwIST method [43], which splits the matrix into a two-step iterative equation.

The background properties are calculated and updated at each DBIM iteration with the TwIST solution until the residual error is minimised, and this yields the reconstruction of the dielectric properties of the imaging domain.

The initial model (“starting guess”) for the algorithm is shown in Figure 4a, and consists of a simplified two-dimensional (2D) representation of the experimental setup, which includes the tank filled with the matching medium and an ellipsoid that mimics the average brain tissue with dimensions of 153 × 112 mm. These represent the actual size of the phantom’s axial slice at the height where the antennas are located. The algorithm simplifies the system’s antennas with line sources, which are placed at the same locations as the experiment. To calibrate the simulated initial model, we use a “no target” reference measurement, as in our previous work (e.g., see Reference [35]). This calibration method relies on the signal difference between the experimental and the simulated “no target” scenarios, to calibrate the signals received from the “target” experiment. It is used to eliminate the errors induced by the differences between the simulated forward model and the experiment.

We must note that an exact “no target” reference measurement will not be available in a realistic clinical scenario such as stroke detection. Aside from an “empty tank” measurement which could always be used as reference, one could also use a reference signal measured from an “average homogeneous head phantom” taken from a set of phantoms with different properties, following the approach used for imaging numerical breast phantoms in Reference [32], for example. This approach, however, would still provide the algorithm with very different signals from the true “no target” signals in stroke detection, as the brain structure is much more complex than any “homogeneous average brain” phantom (see also the Discussion section), and a more complex, in-homogeneous reference phantom may be better suited for data calibration. We also note that our DBIM-TwIST approach has been shown to be capable of reconstructing accurately complex in-homogeneities using simulation data from complex numerical breast phantoms [32], but the problem of detecting the stroke inside the complex yet unknown brain structure may be more challenging. We partially examine this issue in Section 3.2, by using a “no target” scenario from a “day 1 phantom” and a target scenario from a “day 6 phantom” with almost 20% higher dielectric properties. The issue will be fully examined in our future work and presents, arguably, the most significant obstacle that MWT methods need to overcome prior to their clinical use.

To take into account the materials’ dispersive dielectric properties, we employ a first-order Debye model for the average brain phantom and the 90% glycerol-water mixture:

(4) $$\begin{array}{c} \epsilon_{s}\left(\omega \right)=\epsilon_{\infty }+\frac{\Delta \epsilon }{1+j\omega \tau }+j\frac{\sigma_{s}}{\omega \epsilon_{0}}, \end{array}$$

where $\epsilon_{\infty }$, Δϵ and $\sigma_{s}$ are the parameters of the single pole Debye model provided in Table 3. We use the parameters of Table 3 together with a forward solver based on the finite-difference time-domain (FDTD) method to calculate the scattered and background fields, and then apply DBIM-TwIST to reconstruct the dielectric properties inside the imaging domain with a grid voxel size of 2 mm. The complex permittivity values in (2) are replaced by the frequency-dependent Debye model of (4) and are updated at each DBIM iteration, thus are used to update the contrast function. The reconstructed results present estimated real and imaginary parts of permittivity which are calculated from the updated Debye models at each frequency [23]. Moreover, we perform the same number of 15 DBIM-TwIST iterations at each frequency for both single and multiple frequency reconstructions attempted in this study. The running time for each DBIM iteration was approximate 8 seconds using MATLAB R2019b run on an Intel i7 processor with 16 GB RAM memory, leading to an execution time of 2.5 min for 15 iterations of DBIM at each frequency. By means of an example, the total execution time for the case of the h-stroke with one layer and frequency hopping between 0.5 and 1.5 GHz is 24 min.

## 3. Results

In our first set of experiments in Section 3.1, our aim is to show our method’s potential to determine the type of stroke from estimating its dielectric properties. To this end, we examine two phantoms of average brain tissue in which we inserted a target emulating h- and i-stroke, respectively. In the second set of experiments in Section 3.2, our aim is to test detection performance in cases where the inverse experimental model is slightly different than our forward model. To this end, we examine two additional phantoms with h-stroke targets. The first one is a two-layer phantom including a thin CSF-mimicking layer, and the second one is a brain phantom which we let it set for 6 days before conducting the “target” measurements, so that its properties are different in comparison with the first day when the “no-target” measurements were taken.

### 3.1. Detection and Classification of Stroke Targets

**Figure 5:**
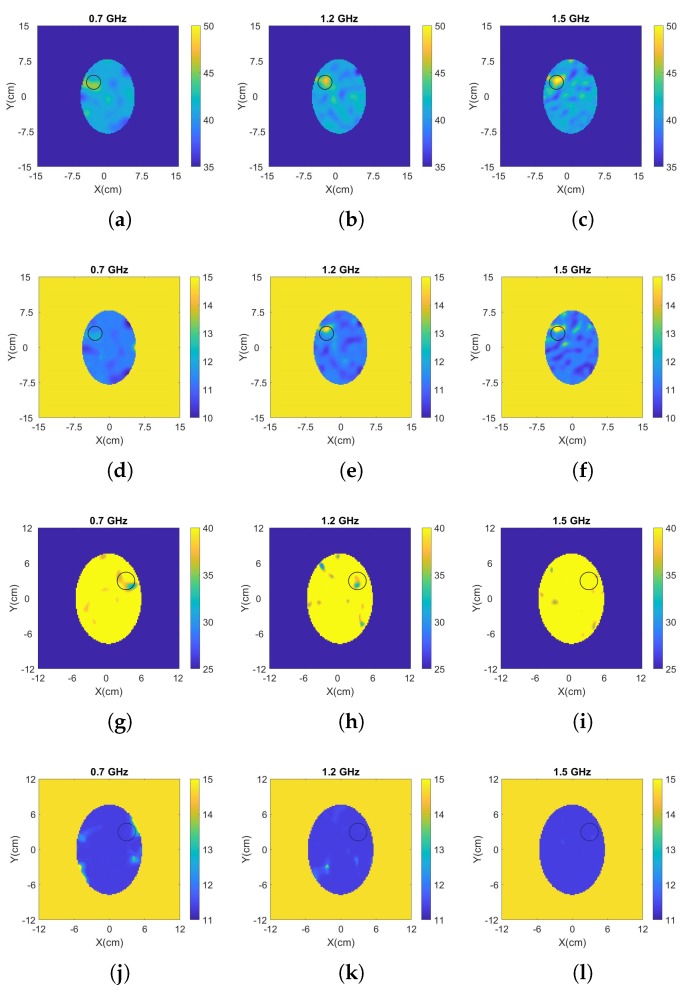

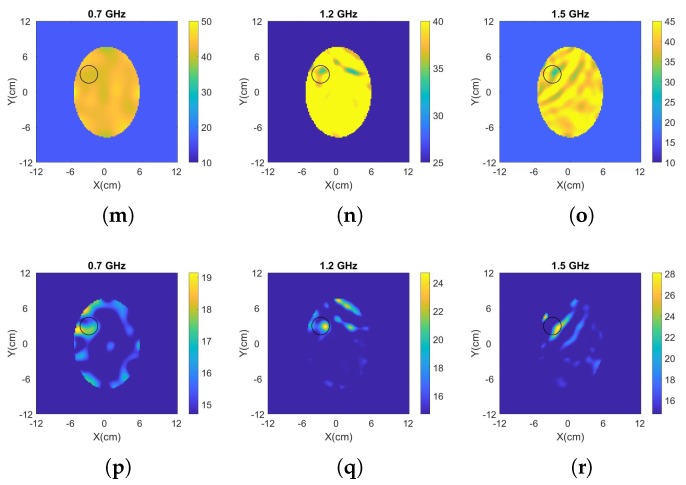
Results from single frequency reconstructions of the real (first, third and fifth line) and imaginary (second, fourth and sixth line) part of the complex permittivity for: (**a–f**) h-stroke, (**g–l**) 25% i-stroke and (**m–r**) 50% i-stroke.

**Figure 6:**
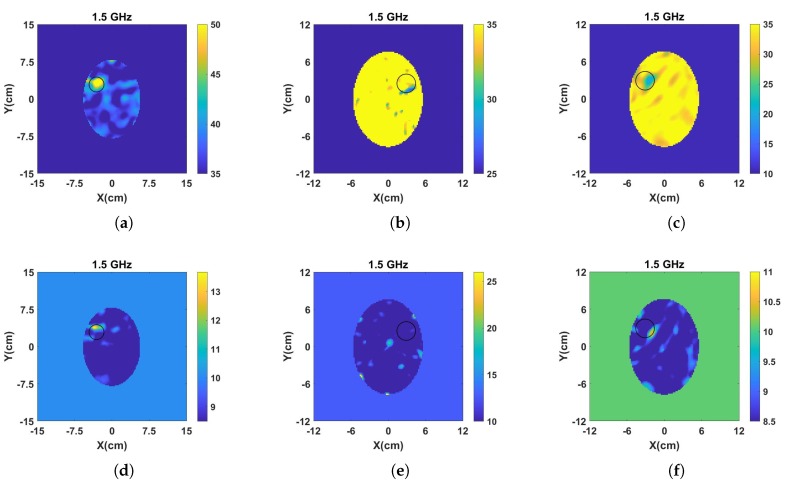
Reconstructed: (**a–c**) real and, (**d–f**) imaginary part of the complex permittivity for h-stroke (left), 25% i-stroke (middle) and 50% i-stroke (right). The complex permittivity was calculated at 1.5 GHz using the frequency hopping approach in a frequency range of 0.7–1.5 GHz.

Figure 5 and Figure 6 present the reconstructed real and imaginary parts of the complex permittivity for the phantom inside the prototype of Figure 4b containing h-stroke and i-stroke targets as well as a target which has 50% lower dielectric constant than the average brain phantom as an extreme case (50% i-stroke). Images illustrate the whole tank, however, the reconstruction domain contains only the brain ellipsoid inside the antenna array. Representative single-frequency reconstructions in the range 0.7–1.5 GHz are shown in Figure 5, while results from a frequency hopping approach in the same range are shown in Figure 6. These images suggest that the dielectric constant (real part) of 25% i-stroke is detectable at low frequencies albeit with more artefacts in the images, whilst the h-stroke and the 50% i-stroke, is clearly visible in frequencies above 1.1 GHz for both real and imaginary parts. As the dielectric contrast between the constructed 25% i-stroke and brain phantoms is lower than that of h-stroke and 50% i-stroke, detecting the 25% i-stroke target is more challenging than the other cases, with unsuccessful results for the imaginary part of the complex permittivity. The most encouraging observation from these figures, however, is that there is a clear distinction in the estimation of the dielectric properties for all the three targets: the frequency hopping results of Figure 6 estimate the value of real permittivity $\epsilon^{\prime }$ at 1.5 GHz as $\epsilon^{\prime }$ = 33.8, $\epsilon^{\prime }$ = 20.9 and $\epsilon^{\prime }$ = 56.25 for 25% i-stroke, 50% i-stroke and h-stroke, respectively.

### 3.2. Stroke Target Detection for Brain Phantoms with Unknown Properties

**Figure 7:**
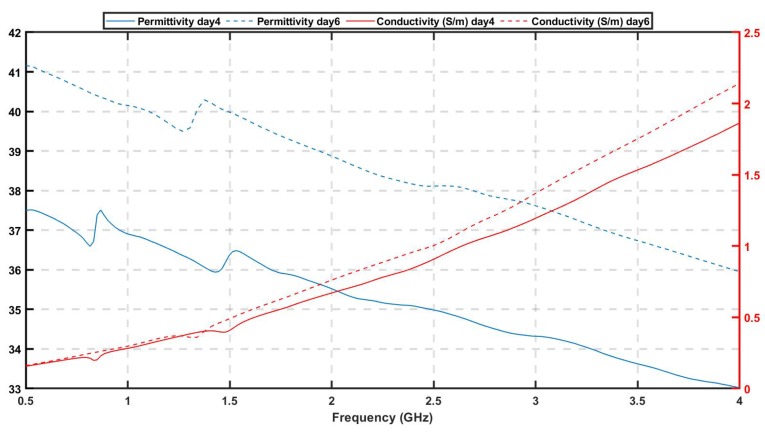
Dielectric properties of brain mimicking phantom at day 4 and day 6 after its preparation.

To move towards more realistic imaging scenarios where the brain’s distribution of tissues will be more complex and their dielectric properties unknown, we have evaluated experimentally the robustness of DBIM-TwIST when the structure and dielectric properties of the brain phantom are slightly different from the “initial guess” of Figure 4a. To examine the effect of differences in the dielectric properties of the average brain phantom where the target is inserted, we prepared a phantom and conducted “no target” measurements after the phantom had set, and then kept it for a week, in which we recorded the change of its dielectric properties over time. Figure 7 shows our measurements for days 4 and 6, where an increase in the permittivity with time is observed possibly due to increase in water dispersion. We then acquired “target” measurements for h-stroke on day 6, to ensure a change in the phantom’s dielectric properties from the “no-target” measurements on day 1. In addition to this test, we also performed experiments with a two-layer phantom (CSF and average brain). To this end, we added a CSF layer of 5 mm thickness using the process described in Figure 3, and with dielectric properties plotted in Figure 2.

**Figure 8:**
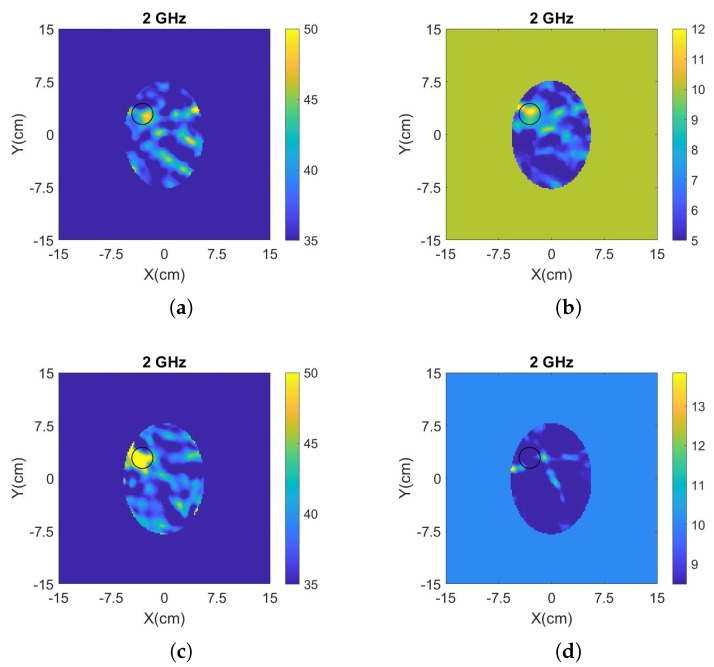
(**a**) Reconstructed real and (**b**) imaginary part of the complex permittivity for the phantom inside the tank of Figure 4b, when “no-target” and “h-stroke target” measurements were conducted at day 1 and day 6 after the preparation of phantom, respectively. (**c**) Reconstructed real and (**d**) maginary part of the complex permittivity for a two-layer phantom with an “h-stroke target” and a CSF layer which is unknown to the imaging algorithm. The complex permittivity was calculated at 2 GHz using a frequency hopping approach in 1.1–2 GHz.

Figure 8 presents the reconstructed images of the real and imaginary parts of complex permittivity using the data obtained by the measurements at days 1 and 6 after the preparation of the brain phantom, as well as the reconstructed images of the two-layer phantom including the CSF layer. These reconstructions were produced by frequency hopping in the range of 1.1–2.0 GHz. The values of the reconstructed real and imaginary parts for the h-stroke target at 2 GHz are $\epsilon^{\prime }$ = 49.28, $\epsilon^{\prime \prime }$ = 12.19 for the one-layer phantom, and $\epsilon^{\prime }$ = 62.38, $\epsilon^{\prime \prime }$ = 8.48 for the two-layer phantom, respectively. With the exception of the imaginary part for the two-layer phantom, these images provide a clear indication of where the h-stroke is located and estimate its dielectric constant with satisfactory accuracy. These reconstructions confirm that the algorithm is sufficiently robust to detect and localize the target successfully in cases with “mild” uncertainties in the true background medium in the experiment and its “initial guess” in the imaging algorithm. We note that we have also performed single frequency reconstructions for both cases (not shown here), which indicate that best results are obtained in the 1.1–1.6 GHz range, which agrees with the operating frequencies of the antennas employed in our measurements [42].

## 4. Discussion

We presented an initial experimental assessment of a microwave tomography prototype based on the DBIM-TwIST algorithm for brain stroke detection and classification. This MWT system was able to differentiate between targets that mimic haemorrhagic and ischaemic stroke, based on the difference in their estimated dielectric properties. Moreover, the system was able to reconstruct the target inside a brain phantom even when its structure or dielectric properties are different from the “initial guess” used in the inversion. These results benefited from reconstructions in multiple frequencies based on the use of our in-house developed spear-shaped antennas which operate in a wide range from 0.5 GHz to 2.5 GHz. Moreover, the flexible tuning of the DBIM-TwIST parameters allow an easy adaptation of the algorithm to inverse problem at hand. We note that, as with most non-linear inverse methods, the DBIM-TwIST parameters must be tuned based on considerations that relate to both the imaging problem and the experimental prototype. Moreover, experimental measurement errors may result in certain frequencies providing more accurate reconstructions than others. In the past, we have proposed a method to discard data dominated by measurement error, based on a correlation technique [35]. Regardless of whether such a pre-processing step is applied to the measured data, frequency hopping is an excellent way to increase the accuracy and robustness of the algorithm [32].

Moreover, our previous work in Reference [32] has confirmed that the DBIM-TwIST (as any other DBIM implementation) is very sensitive to the initial guess of the inverse model. While this has been partially addressed in this work with our investigation in Section 3.2, it will become a much more challenging problem in a realistic brain stroke detection application, where the structure of the brain is much more complex than a homogeneous average brain phantom.

We also note that our experimental validation required that the antenna array is placed at the same height as the target. We are currently working on a more realistic experimental validation process which involves acquiring data for more than one height and employing a three-dimensional (3D) version of the DBIM-TwIST.

The experimental setup contains a tank filled with matching liquid in which we immersed the antennas and the phantoms. However, our current and future work is focused on designing and proposing a portable helmet which will incorporate the matching medium and the antennas. Algorithm-wise, the image reconstruction can be accelerated by frequency selection [35] and a more accurate initial guess which will also increase the stability of the algorithm. However, knowing a priori information for brain imaging is very challenging. For this reason, we have assumed that the initial model is filled with homogeneous average brain tissue. In Reference [32] a method was proposed to estimate the optimal initial guess based on the residual error of different known cases, which we will explore in our future work.

While there are a few experimental studies on detecting a blood-like target using microwave imaging methods [15,16,17,44,45], only a few studies exist that demonstrate experimentally the detection of an ischemic-like area. Our results in this respect are encouraging, as they indicate that a classification of the stroke type is possible with a microwave imaging system without the need of training data [18]. This has also been suggested in References [19,46] but with a very different, single-frequency MWT system. This confirms that MWT systems have the potential to be used for early differentiation between i- and h-stroke, which is vital for improving stroke treatment outcomes [47].

We note that the experimental phantoms in this study are oversimplified in relation to the true head and brain anatomy. However, this simplification is necessary to show that our system and algorithm have the potential to differentiate between the two different stroke types based on a quantitative estimation of their dielectric properties. We must acknowledge, however, that the imaging performance of the algorithm and prototype depends strongly on the complexity of the brain, and hence, the experimental phantom that represents it. The skin and skull, for example, introduce strong scattering layers, and the distribution of the CSF can be complex and can obscure the signal from the target. While our previous work has shown that tools such as frequency hopping and optimization of the initial guess can have a significant positive impact, for example in reconstructing breast compositions in the presence of an unknown skin layer [32], application of our approach to a complex brain phantom may require prior information of the phantom’s anatomical structure. This in practice could be acquired from average numerical brain models and a careful calibration procedure. These issues are critical to provide a more informed answer to whether MWT can be clinically attractive as a stroke detection method and will be investigated in our future work.

We also note that we are currently on the next version of our head phantom, which will feature a more complex structure with various tissue-mimicking materials resembling different brain tissue layers, as well as outer layers such as the skull and the skin. Moreover, in our future work we will use moulds in realistic head shape that will allow us to include a wig to take into account the impact of hair. As shown in Reference [48], a wig’s dielectric properties are very similar to the dielectric properties of natural hair, therefore, a wig can be easily used as an alternative for those experiments. Our successful reconstructions for the phantom with an added thin elliptical CSF layer, although not fully realistic, are a step forward towards our goal of successful detection in complex head phantoms.

Our future work will also focus on validating the proposed MWT approach further towards the development of a portable microwave head scanner designed for stroke detection, which will apply a multiple-frequency 3D DBIM-TwIST algorithm for imaging. This will include assessing the capabilities of our MWT system with phantoms with increased complexity resembling the actual anatomy and dielectric properties of the brain. We will also examine other applications of our MWT approach such as lymph node detection for breast cancer.

## Figures and Tables

**Table 1:** Concentrations of materials used for 100 ml of tissue mimicking phantoms.

Phantom Type	Water	Gelatine Powder	Kerosene	Safflower Oil	Propanol	Surfactant
Average brain	60 mL	11 gr	13 gr	13 gr	2.5 mL	1.5 mL
CSF/Blood	80 mL	16 gr	-	-	4 mL	-
Ischemia	50 mL	8.5 gr	20 gr	20 gr	1.5 mL	1.5 mL

**Table 2:** Dielectric properties of brain phantoms over time at 1 GHz.

Measured Property	Day 1	Day 6
$\epsilon^{\prime }$ sample 1	44.5	51.2
$\epsilon^{\prime }$ sample 2	46.2	51.6
$\epsilon^{\prime \prime }$ sample 1	0.43	0.51
$\epsilon^{\prime \prime }$ sample 2	0.42	0.48

**Table 3:** Debye parameters of materials after curve fitting to measured dielectric properties.

Material Type	Δϵ	$\epsilon_{\infty }$	$\sigma_{s}$
90% glycerol-water	6.56	16.86	0.3232
Average brain	30	10	0.147

## Abbreviations

The following abbreviations are used in this manuscript:

ABS	Acrylonitrile Butadiene Styrene
CGLS	Conjugate Gradient method for Least Squares
CSF	Cerebrospinal fluid
CT	Computed Tomography
EM	Electromagnetic
DBIM	Distorted Born Iterative Method
FDTD	Finite-Difference Time-Domain
IST	Iterative Shrinkage/Thresholding
MWI	Microwave Imaging
MWT	Microwave Tomography
MRI	Magnetic Resonance Imaging
TwIST	Two-step Iterative Shrinkage Thresholding
VNA	Vector Network Analyzer
2D	Two-dimensional
3D	Three-dimensional
